# Anemia, transfusion, and phlebotomy practices in critically ill patients with prolonged ICU length of stay: a cohort study

**DOI:** 10.1186/cc5054

**Published:** 2006-09-26

**Authors:** Clarence Chant, Gail Wilson, Jan O Friedrich

**Affiliations:** 1 Leslie Dan Faculty of Pharmacy, University of Toronto, 144 College Street, Toronto, Ontario, Canada M5S 3M2; 2 Specialized Complex Care Program, St. Michael's Hospital, 30 Bond Street, Toronto, Ontario, Canada M5B 1W8; 3 Interdepartmental Division of Critical Care, University of Toronto, and Critical Care and Medicine Departments, St. Michael's Hospital, 30 Bond Street, Toronto, Ontario, Canada M5B 1W8

## Abstract

**Introduction:**

Anemia among the critically ill has been described in patients with short to medium length of stay (LOS) in the intensive care unit (ICU), but it has not been described in long-stay ICU patients. This study was performed to characterize anemia, transfusion, and phlebotomy practices in patients with prolonged ICU LOS.

**Methods:**

We conducted a retrospective chart review of consecutive patients admitted to a medical-surgical ICU in a tertiary care university hospital over three years; patients included had a continuous LOS in the ICU of 30 days or longer. Information on transfusion, phlebotomy, and outcomes were collected daily from days 22 to 112 of the ICU stay.

**Results:**

A total of 155 patients were enrolled. The mean age, admission Acute Physiology and Chronic Health Evaluation II score, and median ICU LOS were 62.3 ± 16.3 years, 23 ± 8, and 49 days (interquartile range 36–70 days), respectively. Mean hemoglobin remained stable at 9.4 ± 1.4 g/dl from day 7 onward. Mean daily phlebotomy volume was 13.3 ± 7.3 ml, and 62% of patients received a mean of 3.4 ± 5.3 units of packed red blood cells at a mean hemoglobin trigger of 7.7 ± 0.9 g/dl after day 21. Transfused patients had significantly greater acuity of illness, phlebotomy volumes, ICU LOS and mortality, and had a lower hemoglobin than did those who were not transfused. Multivariate logistic regression analysis identified the following as independently associated with the likelihood of requiring transfusion in nonbleeding patients: baseline hemoglobin, daily phlebotomy volume, ICU LOS, and erythropoietin therapy (used almost exclusively in dialysis dependent renal failure in this cohort of patients). Small increases in average phlebotomy (3.5 ml/day, 95% confidence interval 2.4–6.8 ml/day) were associated with a doubling in the odds of being transfused after day 21.

**Conclusion:**

Anemia, phlebotomy, and transfusions, despite low hemoglobin triggers, are common in ICU patients long after admission. Small decreases in phlebotomy volume are associated with significantly reduced transfusion requirements in patients with prolonged ICU LOS.

## Introduction

Anemia of critical illness is a common problem in patients admitted to the intensive care unit (ICU) [1-5]. The cause of anemia is likely multifactorial, but frequent phlebotomy has been cited as a contributing factor, resulting in frequent prescription of packed red blood cell (PRBC) transfusions [1,4]. Current evidence suggests that PRBC transfusions are associated with infectious and inflammatory complications and transfusion errors, and their routine use does not result in improved patient outcomes in a variety of indications [6-8]. Hemoglobin levels and transfusion practices have been well characterized by recent epidemiologic studies [2,3,5,9,10] in ICU patients with short-to-moderate length of stay (LOS). However, in patients with very long LOS (≥30 days) because of prolonged need for life support therapies, such data are unavailable. In contrast to other ICU patients, these long-stay patients have usually overcome their initial reason for admission and face different issues such as secondary infections and complications caused by the prolonged immobility associated with weaning from life support therapies.

Despite being a relatively small proportion of total admissions to the ICU, these patients nevertheless consume a disproportionately large amount of limited resources for their care [11-13]. Thus, it is important to appreciate this frequent problem in this distinct and resource intensive subgroup of ICU patients. We therefore conducted a cohort study to characterize the frequency of anemia, phlebotomy usage, and transfusion practices in patients with prolonged ICU stay, and to determine factors associated with PRBC transfusion.

## Materials and methods

### Data sources

This was a retrospective, single center, observational cohort study conducted in a 24-bed, closed medical-surgical ICU at St. Michael's Hospital, which is a tertiary care center affiliated with the University of Toronto. Patients requiring mechanical ventilation or intense physiologic support or monitoring were admitted to the ICU and cared for by a multidisciplinary health care team under the direction of an attending intensive care physician. All patient care decisions were made independent of data collection. During the period of study, there was no standardized protocol for transfusion or phlebotomy practice.

### Data collection

All patients admitted to the ICU between 1 January 2001 and 31 December 2003 with a continuous LOS of 30 days or longer were enrolled in the study. Each patient's medical records and electronic laboratory database files were used to obtain information pertaining to baseline (at ICU admission) demographics, pre-existing comorbidities, admission Acute Physiology and Chronic Health Evaluation (APACHE) II score [14], use of iron and erythropoietin, and weekly hemoglobin indices to day 21. Patients admitted with pre-existing diagnoses of hypothyroidism (13 patients) or vitamin B_12 _deficiency (four patients) were continued on their maintenance thyroid or vitamin B_12 _replacement therapies. No patients had documented thyroid, vitamin B_12_, or folate deficiency (potential contributors to anemia) throughout the study period, as indicated by normal serum markers whenever these were obtained by clinicians.

In these patients, who had a LOS of 30 or more days in the ICU, daily data collection began on day 21 to ensure that each patient would contribute at least 10 days of data. After day 21, the following data were recorded until ICU discharge to a maximum of 90 days: any need for life support therapies (mechanical ventilation, inotropes or vasopressors, or acute renal failure requiring renal replacement therapy), daily Sequential Organ Failure Assessment (SOFA) scores [15], daily hemoglobin values (if measured), estimated daily phlebotomy volumes (see below), and any blood transfusions. For all PRBC transfusions given, information regarding the reason and transfusion trigger was documented by examining the pertinent clinical data and physician/nursing documentation. When no apparent indications or clinical status changes (for instance, hemodynamic instability, active ischemia, or failed ventilator weaning) or falling hemoglobin values were documented, the transfusion indication was deemed to be 'none'. A 'bleeding' transfusion indication was assigned if this was documented in the chart or if the transfusion was administered in association with a predefined hemoglobin decrease of more than 2.0 g/dl over 24 hours. Each transfusion event, which may involve one or more units of PRBC, was assigned one dominant reason for transfusion by one of the investigators. Each patient may have more than one transfusion event per day.

Daily phlebotomy volumes after day 21 were estimated based on the number of tests ordered. To calculate the volume of blood drawn from the patients, we used an average value for each type of test for each vial of blood (arterial blood gas = 2 ml, chemistry = 5 ml, coagulation = 4.5 ml, complete blood count = 5 ml, blood culture = 10 ml, and drug level/miscellaneous = 5 ml) along with a standard amount for discard (2 ml) in between blood samples, based on the current standard nursing practice in the ICU.

Finally, outcome variables including ICU and hospital LOS, and ICU mortality were recorded. For patients with multiple ICU admissions, only data from the first ICU admission that met the inclusion criteria were used. The study was approved by the hospital's institutional review board and a waiver of consent was granted.

### Statistical analysis

Continuous variables are summarized as mean ± standard deviation or median (interquartile range) for normally and non-normally distributed variables, respectively. Comparisons between transfused and never transfused patients who did not have active bleeding were conducted using Student's *t *test or Wilcoxon's test for normally and non-normally distributed continuous variables, respectively. χ^2 ^or Fisher's exact test were used to compare categorical variables.

For patients who did not have active bleeding, all variables recorded above were entered into univariate logistic regression analyses to determine factors associated with any PRBC transfusions. All variables with a *P *value < 0.20 by univariate logistic regression analysis were entered into a multivariate logistic regression model using backward selection. Variables with a *P *value < 0.10 were retained in the multivariate model. We report odds ratio (ORs) and 95% confidence intervals (CIs), and interpret two-sided *P *values < 0.05 as statistically significant. Logistic regression (transfused versus not transfused) was used as the primary analysis because most nonbleeding patients who received a blood transfusion were transfused with relatively few units of blood. Multivariate linear regression, using the number of units of PRBC transfused as the dependent variable, and a similar backward selection technique to eliminate nonsignificant variables, was carried out as a secondary analysis. All statistical calculations were conducted using SAS version 8.2 (SAS Institute Inc., Cary, NC, USA). Because the discard volume required estimation, sensitivity analyses on the regression models using discard volumes of 0 (no wastage) and 7 ml were also performed. Seven milliliters was chosen as the upper limit of discard volume because this was the maximum volume of the phlebotomy tubes (used by some ICU nurses as discard).

## Results

**Figure 1:**
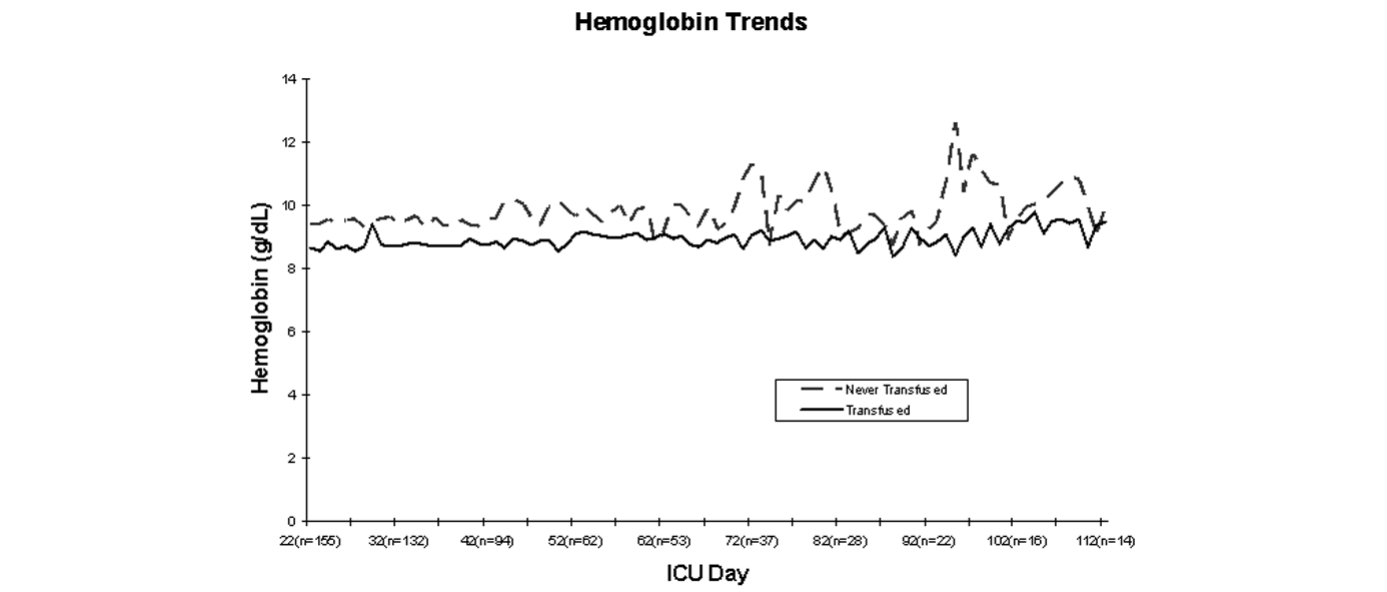
Hemoglobin trend over time by transfusion status. ICU, intensive care unit.

Over the 3-year study period, 170 patients had an ICU LOS of 30 days or longer, representing 5.4% of the total 3172 ICU admissions. Five patients who had ICU LOS longer than 21 days in other hospitals before transfer to the study hospital and 10 patients for whom phlebotomy and transfusion data were unavailable were excluded. Baseline characteristics at ICU admission and clinical outcomes for the remaining 155 patients are summarized in Tables 1 and 2. The patients included in the cohort had an average age of 62 ± 16 years, moderate severity of illness, multiple pre-existing comorbidities, and relatively stable mean daily SOFA scores, which on average declined by approximately 1 point between day 21 and ICU discharge. ICU admission hemoglobin was 11.1 ± 2.5 g/dl, which is well below the lower limit of normal for our laboratory (13.0 g/dl). Table 1 describes weekly hemoglobin values up to day 21. Figure 1 shows daily hemoglobin values for all patients over time after day 21 separated by transfusion status. It appears that the hemoglobin values reach a 'steady state' of around 9.0 g/dl by days 7–14 (Table 1 and Figure 1).

**Figure 2:**
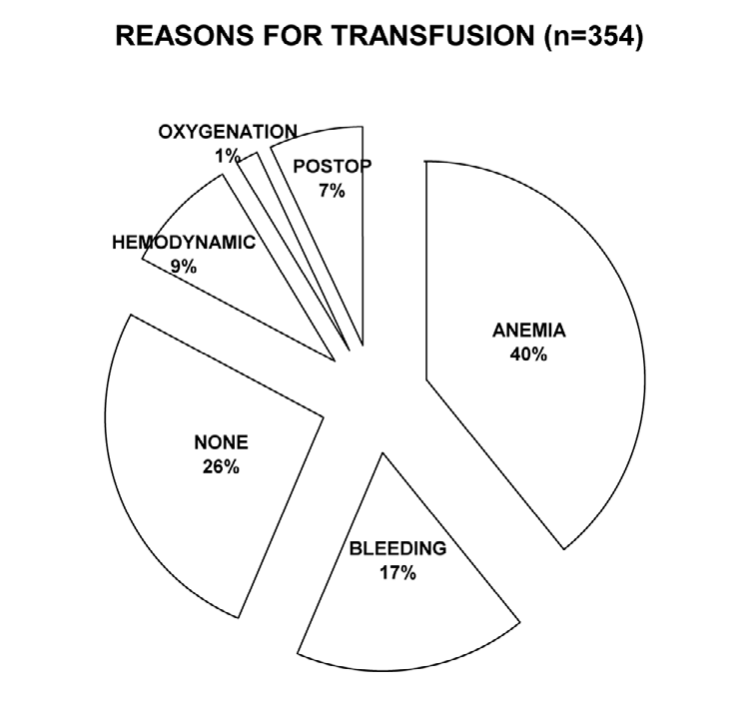
Reasons for transfusions.

**Figure 3:**
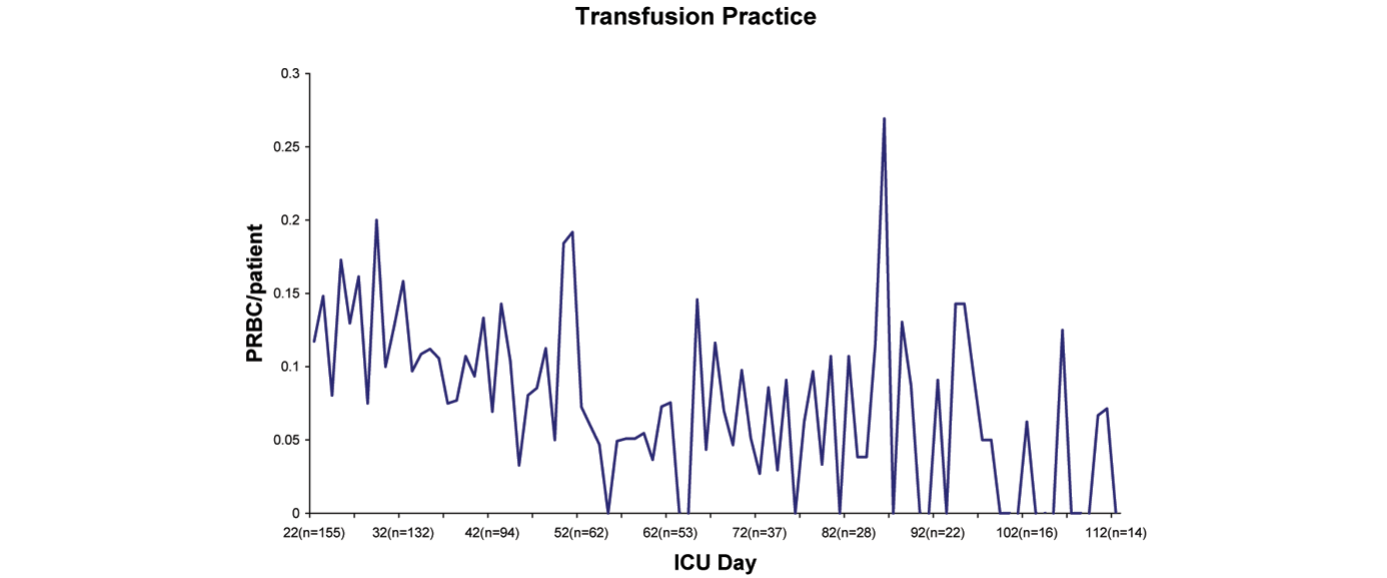
Transfusion over time. ICU, intensive care unit; PRBC, packed red blood cells.

Ninety-six of the 155 (62%) patients were transfused with 1 or more units of PRBC after day 21. A total of 542 units of PRBC (median [interquartile range] = 1 [0–4] unit/patient) were transfused on 354 separate occasions. The majority (81%) of the transfusions was administered between ICU days 22 and 57, with the remainder occurring after day 57. These transfusions were given at a mean hemoglobin trigger of 7.7 ± 0.9 g/dl (range 5.1–10.7 g/dl), and 73% of the transfusions were given at a trigger above 70 g/l. Reasons for transfusion after day 21 were as follows: active bleeding (17%), low hemoglobin concentration (40%), postoperative resuscitation (7%), to improve oxygenation or hemodynamic status (10%), and no identifiable reason (26%; Figure 2). Although the total number of units of PRBC transfused decreased over time, the number per patient (mean = 0.075 ± 0.056 units/patient per day, which is equivalent to 0.051 ± 0.034 transfusion events/patient per day or 5.1 ± 3.4% of patients transfused daily) remained fairly constant, especially by around days 50–60 (Figure 3).

**Figure 4:**
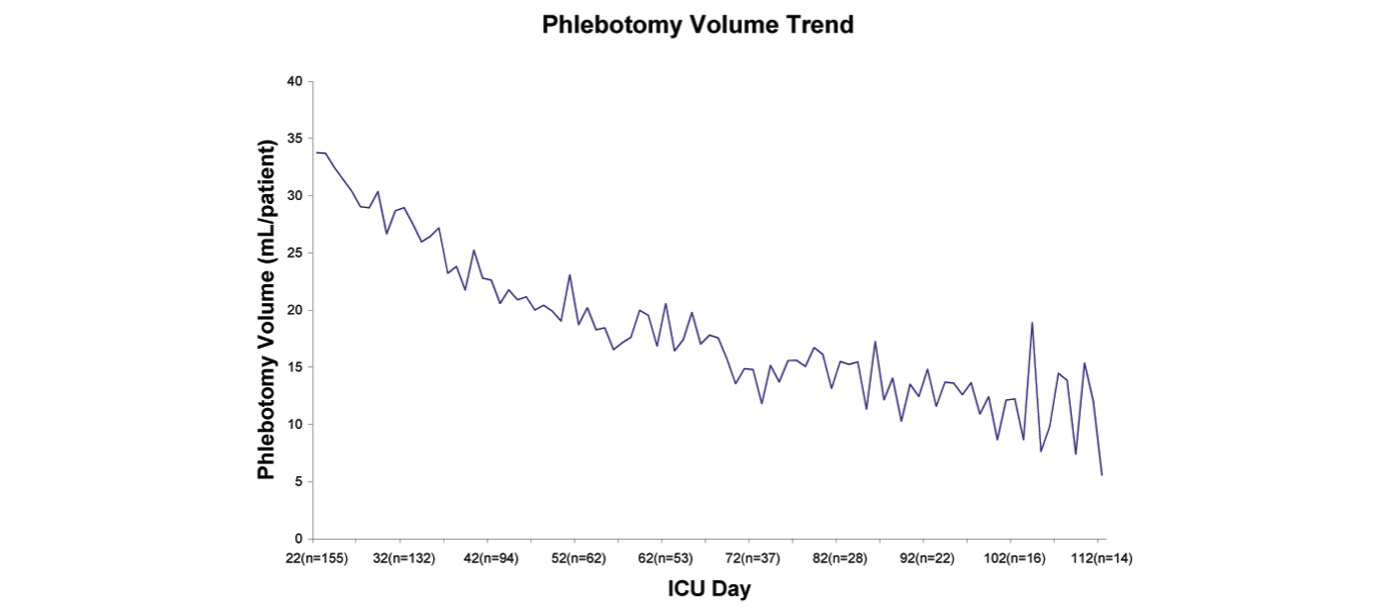
Phlebotomy volume over time. ICU, intensive care unit.

**Figure 5:**
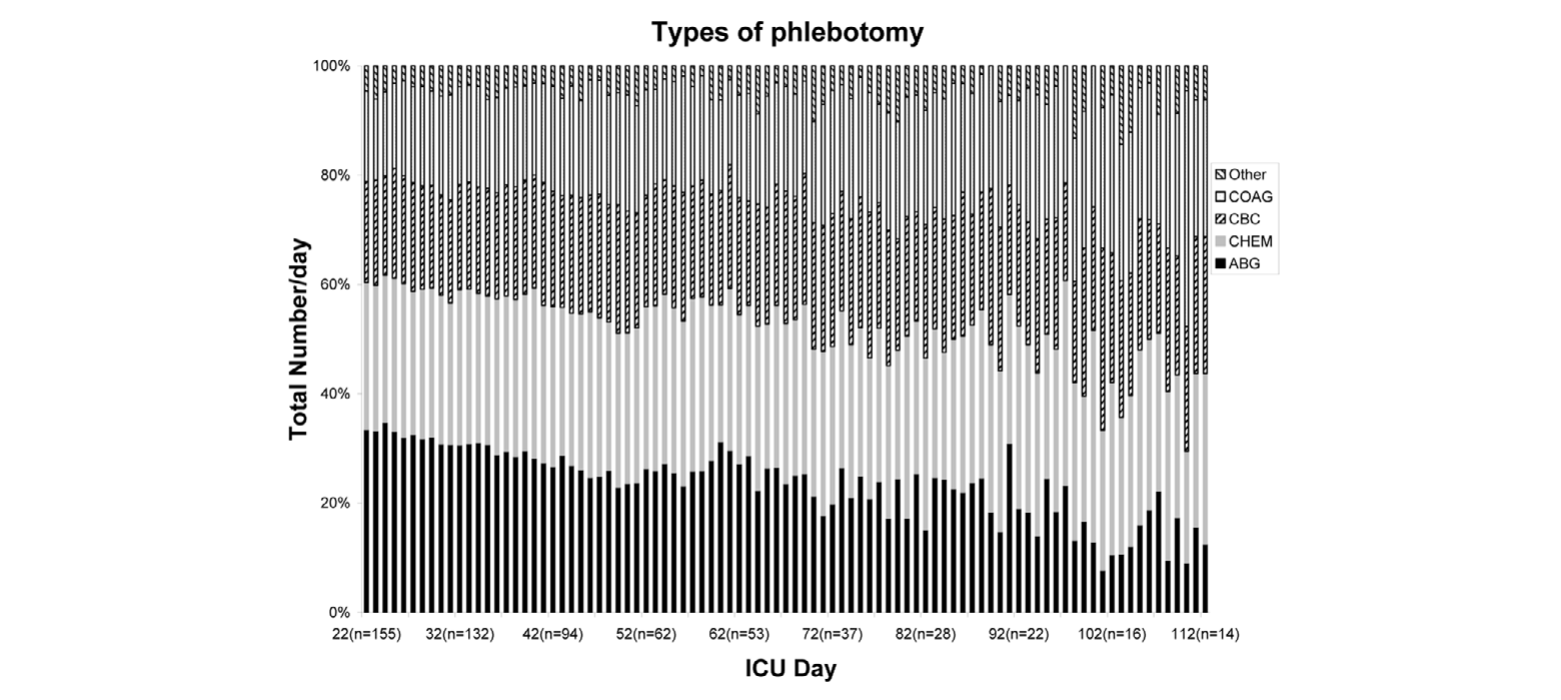
Types of phlebotomy over time. Figures in parenthesis on the x-axis denote the number of patients. ABG, arterial blood gas; CBC, complete blood count; CHEM, chemistry; COAG, coagulation; Other, blood cultures and others.

A significant number of phlebotomy procedures were conducted in this cohort of ICU patients after day 21. A total of 129 l blood was phlebotomized over 9,191 patient-days. An average of 13.3 ± 7.3 ml/day was phlebotomized after day 21, and 3.8 ± 1.5 vials of blood/patient per day were obtained. The amount of blood phlebotomized each day in the entire cohort decreased as the LOS increased (Figure 4). The largest group of tests was arterial blood gas, followed by chemistry profile, complete blood count, and coagulation profile (Figure 5). Although the proportion of chemistry profiles, complete blood count, and coagulation profiles remained fairly constant over time, the amount of arterial blood gas tests decreased.

When 25 patients with active bleeding (and thus a definitive therapeutic reason for PRBC transfusions) were excluded, the remaining 71 patients who received a blood transfusion after day 21 had greater acuity of illness than did nonbleeding patients who never received a blood transfusion after day 21, as reflected by higher admission APACHE II scores, mean daily SOFA scores, requirements for life support therapies, ICU LOS, and mortality (Tables 1 and 2). In addition, nonbleeding patients who received transfusions had lower hemoglobin levels, were more likely to receive iron or erythropoietin, and were more likely to lose more blood to phlebotomy during their ICU stay than were non-bleeding patients who never required a blood transfusion (Tables 1 and 2). Overall, 24 patients (15%) received one or more doses of erythropoietin. Of those, all but one patient received the drug because of anemia associated with acute or chronic kidney disease requiring dialysis, frequently after having required a blood transfusion.

Based on these results, the following variables were entered into the multivariate logistic regression model as dependent variables: the continuous variables of admission APACHE II score, mean daily SOFA score and phlebotomy volumes (both after day 21), and ICU LOS; and the binary variables of sex, surgical admission, coronary artery disease, erythropoietin therapy, iron therapy, day 21 hemoglobin, inotrope/vasopressor therapy, and acute renal failure requiring dialysis. (This was based on the predefined analysis plan to include only variables with *P *< 0.20 on univariate analysis. However, the multivariate results are unchanged when all possible explanatory variables found in Tables 1 and 2 are entered into the regression analyses regardless of the *P *value on univariate analysis.) After day 21, the characteristics independently associated with PRBC transfusion in the nonbleeding patients were as follows: average daily phlebotomy volume while in the ICU (OR 1.22 per incremental ml/day phlebotomized, 95% CI 1.11–1.34; *P *< 0.0001), ICU LOS (OR 1.025 per additional day in ICU, 95% CI 1.006–1.045; *P *= 0.008), baseline (day 21) hemoglobin level (OR 2.1 per 1 g/dl lower hemoglobin, 95% CI 1.4–3.4; *P *= 0.001), and treatment with erythropoietin (OR 6.4, 95% CI 1.2–34.3; *P *= 0.03). Stated in other terms, the odds of being transfused after day 21 in these patients doubled with any of the following: an average 3.5 (95% CI 2.4–6.8) ml/day blood loss to phlebotomy, an additional 28 (95% CI 16–116) days in ICU, or a day 21 hemoglobin level that was 0.9 (95% CI 0.6–2.3) g/dl lower than the average. The area under the receiver operating characteristic curve for this model was 0.86. There were no significant second order interactions among these variables.

Sensitivity analysis using a discard volume of 0–7 ml (base case of 2 ml) resulted in no change to the regression model with respect to the statistically significant predictive risk factors. The ORs for average daily phlebotomy were 1.27 (95% CI 1.13–1.42; *P *< 0.0001) and 1.15 (95% CI 1.08–1.23; *P *< 0.0001) per incremental ml/day phlebotomized, assuming a 0 and 7 ml discard volume, respectively. This corresponds to an extra 2.9 (95% CI 2.0–5.7) and 4.9 (95% CI 3.3–9.6) ml/day of additional phlebotomy to double the odds of being transfused after day 21 in these patients, assuming a 0 and 7 mL discard volume, respectively.

Results from the secondary analysis using multivariate linear regression demonstrated that the number of PRBC transfused was significantly and independently associated with daily phlebotomy volume, ICU LOS, and acute renal failure requiring dialysis. After day 21, 1 unit of PRBC was transfused for every additional 2.2 (95% CI 1.7–3.4; *P *= 0.0002) ml blood drawn/day in patients with acute renal failure and 5.4 (95% CI 4.5–8.8; *P *< 0.0001) ml blood drawn/day in patients without acute renal failure. Similarly, 1 unit of PRBC was transfused for every 12 (95% CI 8–24; *P *= 0.004) additional days in ICU in patients with acute renal failure and 45 (95% CI 29–103; *P *= 0.0007) additional days in ICU in patients without acute renal failure.

## Discussion

In our cohort of long-stay ICU patients, phlebotomy, anemia, and transfusions – despite low hemoglobin triggers – are common long after admission. After day 21, phlebotomy volume, ICU LOS, baseline (day 21) hemoglobin, and erythropoietin therapy (used almost exclusively in dialysis-dependent renal failure in our ICU) were independently associated with being transfused and the number of PRBC transfused using multivariate regression analysis.

Our patients' admission hemoglobin of 11 g/dl and its convergence over time to 9.1 g/dl are very similar to that seen across European countries and in North America [2,3]. This highlights the importance of the issue and the potential for ongoing significant resource (for instance, PRBC and erythropoietin) utilization that appear to continue throughout a patient's ICU stay. After patients have overcome their initial episode of critical illness and the underlying cytokines that are at least partly responsible for the pathophysiology of anemia of critical illness have presumably abated, hemoglobin levels do not appear to return to premorbid levels while the patients remain in the ICU. The finding that phlebotomy volume was an independent predictor of transfusion requirements in our patients, even after adjusting for other confounders, suggests that it may contribute to the lack of recovery of hemoglobin levels. Furthermore, our study results suggest that even small reductions in phlebotomy volumes, the only readily modifiable risk factor identified in the study, may significantly reduce the number of PRBC transfusions.

PRBC transfusions were surprisingly common in our patient cohort, even when actively bleeding patients were excluded. Our transfusion rate of 62% (after day 21) is higher than the 20–53% reported in other multicentre studies involving ICU patients studied from the time of ICU admission with very short median LOS [2,3,5,9,10], but it is lower than the 85% observed in patients with a LOS greater than 1 week [4]. Our transfusion trigger of 7.7 g/dl approaches the threshold proposed by the TRICC (Transfusion Requirements in Critical Care) trial [6] of 7.0 g/dL, although significant variability exists. This trigger is slightly lower than was observed in multicentre studies, in which the mean transfusion trigger ranged from 8.2 to 8.6 g/dl [2,3,9,10], but it is similar to that in a more recently reported ICU patient cohort (7.8 g/dl) [5]. This may reflect increasing comfort over time with the lower transfusion threshold suggested by the TRICC trial results. Similar to other studies [2,5,9], the most commonly ascertained reason for transfusion was anemia.

Patients in our cohort had a mean daily phlebotomy volume of 13 ml, which is less than that in previous studies [2-4]. These studies focused on the acute phase of critical illness, in which more diagnostic and monitoring are expected, and reported daily phlebotomy values of 40–70 ml/patient per day. Despite the lower volumes, our patients still averaged almost four vials of blood per day, which is similar to the 4.6 draws per day reported in one of these studies [3]. Therefore, the difference in volume between that study and ours was largely accounted for by the lower volume per test, which was 5 ml in our case and 10 ml in the other. It was surprising to see that our patient cohort, with a median LOS of almost 50 days, was phlebotomized at a frequency similar to that in newly admitted ICU patients with median LOS of 2–4 days [2,3].

The probability of being transfused in this study after day 21 in the ICU was associated with phlebotomy volume, ICU LOS, baseline hemoglobin, and erythropoietin treatment. ICU LOS, baseline hemoglobin, and phlebotomy volumes are intuitive predictors of transfusion requirement, and have been found to be predictors in other studies enrolling patients with considerably shorter LOS [1-4]. Interestingly, severity of illness measures such as APACHE II and mean daily SOFA scores were not predictors; this is contrary to the findings of some [1-3] but not all [5] shorter stay studies. There are a number of possible reasons for this finding. First, APACHE II scores calculated at ICU admission have been shown to be less predictive of outcome in patients whose stays are prolonged [16], as in our study. Second, although complete data were available for all patients for almost all of the variables, daily SOFA scores were unavailable for 24 nonbleeding patients in the present study. Because SOFA scores were correlated with the other predictor variables in the regression model, this lack of complete data might have contributed to a lack of significant additional predictive power of this variable in the final overall regression model. In fact, it is the additional phlebotomy, which may or may not be due to worsening illness (and thus a higher SOFA score), that contributes more directly to the resulting anemia.

Erythropoietin therapy was associated with increased probability of being transfused; this appears contrary to the findings of randomized controlled trials showing that its use reduces PRBC transfusions in ICU patients [17,18]. This discrepancy is probably due to local erythropoietin prescribing practices. In our cohort of patients, erythropoietin was newly prescribed almost exclusively in acute renal failure requiring dialysis and transfusion. It was likely for this reason that erythropoietin therapy, rather than the need for dialysis, was more predictive of transfusion requirements in our dataset. This is supported by the results of the secondary analysis using multivariate linear regression, which showed that the number of units transfused was associated with the presence of acute renal failure requiring dialysis. The probable explanation for the association in our study is that renal failure resulted in both increased transfusions and prescription of erythropoietin. Other studies have also correlated acute renal failure with increased blood loss [1], lower hemoglobin levels [5], and increased phlebotomy [19] in the ICU. The shorter stay study with the longest median LOS (about 10 days) [1] also demonstrated a blunted erythropoietin response in ICU patients, which is aggravated by renal failure.

There are several limitations of the present study. First, it was a retrospective review and so our findings might have been confounded by unmeasured factors. Moreover, associations between variables identified with regression analysis do not necessarily provide evidence of causality, as illustrated in the previous paragraph. Second, although phlebotomy and transfusion practices did not significantly change during the data collection period, the study was conducted in a single center without a standardized transfusion protocol and reflects a unique organization, patient population, and process of care. However, the similarities between our data and those of other multicenter studies suggest that our data may have some generalizability to other similar patients with prolonged ICU LOS in other centers. Studying patients with very long stay may also limit generalizability to ICU patients with shorter stays but because our patients were exposed to a relatively long period of phlebotomy in the ICU, it allowed us to demonstrate that even small increases in phlebotomy appear to be associated with increased transfusions. Moreover, if the important variable is total volume of phlebotomized blood, then the study findings may also have some relevance to more acutely ill ICU patients subjected to larger daily phlebotomy volumes despite their shorter and more intense LOS. Finally, although the number of patients enrolled in the study is relatively large, given the rarity of patients with very long ICU stays, it is still a relatively small number from a statistical perspective, which limits the precision of the parameter estimates for the predictors and reduces the power to detect predictors with smaller effects.

## Conclusion

In summary, anemia was universal and PRBC transfusions common after day 21 in our cohort of patients with prolonged ICU LOS. Surprisingly, anemia and transfusion practices are not dissimilar to those observed in multicentre observational studies evaluating shorter stay patients newly admitted to the ICU. Phlebotomy was a significant modifiable predictor of PRBC transfusions in our study and occurred frequently in these patients who had overcome their initial reason for ICU admission. Future quality and patient safety improvements should be directed at modifiable risk factors such as phlebotomy practices and standardization of transfusion, because even small decreases in phlebotomy volumes appear to be associated with significant reductions in the number of PRBC transfusions.

## Key messages

• Anemia, phlebotomy, and transfusions after day 21, despite a low mean transfusion hemoglobin trigger (7.7 g/dl), remained common in ICU patients admitted over a three year period with LOS of 30 days or longer.

• Multivariate regression analysis identified baseline (day 21) hemoglobin, phlebotomy volumes, ICU LOS, and dialysis-dependent renal failure as being independently associated with transfusion requirements.

• Small increases in average phlebotomy (3.5 ml/day, 95% CI 2.4–6.8 ml/day) were associated with a doubling of the odds of being transfused after day 21 in this cohort of patients.

## Abbreviations

APACHE = Acute Physiology and Chronic Health Evaluation; CI = confidence interval; ICU = intensive care unit; LOS = length of stay; OR = odds ratio; PRBC = packed red blood cells; SOFA = sequential organ failure assessment.

## Competing interests

The authors declare that they have no competing interests.

## Authors' contributions

CC was involved in the conception and design of the study, data acquisition, analysis, and interpretation, and wrote the first draft of the manuscript. GW was involved in the conception of the study, data acquisition and interpretation, and provided critical review of the intellectual content of the manuscript. JF was involved in the conception and design of the study, data acquisition, analysis, and interpretation, and provided critical review of the intellectual content of the manuscript. All authors read and approved the final version of the manuscript.

## Figures and Tables

**Table 1:** Baseline demographic information

Parameter	All patients (*n *= 155)	Never transfused (*n *= 59)	Ever transfused^a ^(*n *= 71)	*P *value
Age (years)	62.3 ± 16.3	61.2 ± 18.0	62.9 ± 15.8	0.56
Male (*n *[%])	98 (63%)	35 (59%)	50 (70%)	0.19
BMI^b ^(kg/m^2^)	26.6 ± 7.3	26.6 ± 7.1	26.5 ± 7.8	0.95
Surgical (%)	64 (41%)	19 (32%)	33 (46%)	0.10
APACHE II score	23.4 ± 8.0	20.5 ± 8.9	24.4 ± 6.9	0.007
Admission diagnosis (*n *[%])				0.24^c^
Respiratory	58 (37%)	26 (44%)	25 (35%)	
Cardiovascular	41 (26%)	14 (24%)	20 (28%)	
Gastrointestinal	32 (21%)	8 (13%)	19 (27%)	
Neurologic	12 (8%)	7 (12%)	4 (6%)	
Other	12 (8%)	4 (7%)	3 (4%)	
Comorbidities (*n *[%])				
CAD	37 (24%)	10 (17%)	19 (27%)	0.18
CHF	18 (12%)	5 (8%)	9 (13%)	0.44
CVA	15 (10%)	7 (12%)	6 (8%)	0.52
DM	31 (20%)	10 (17%)	16 (23%)	0.43
COPD	38 (25%)	15 (25%)	18 (25%)	0.99
HTN	41 (26%)	13 (22%)	20 (28%)	0.42
Cancer	19 (12%)	6 (10%)	11 (15%)	0.37
ESRD	7 (5%)	1 (2%)	5 (7%)	0.22^c^
Medications (*n *[%])				
Erythropoietin	24 (15%)	2 (3%)	16 (23%)	0.002
Iron	39 (25%)	9 (15%)	24 (34%)	0.016
Hemoglobin (g/dl)				
Day 0	11.1 ± 2.5	11.8 ± 2.5	10.8 ± 2.5	0.04
Day 7	9.4 ± 1.4	9.8 ± 1.5	9.2 ± 1.2	0.003
Day 14	9.0 ± 1.2	9.4 ± 1.3	8.8 ± 1.0	0.002
Day 21	9.0 ± 1.1	9.4 ± 1.2	8.8 ± 0.9	0.001

^a^Excludes 25 patients with active bleeding. ^b^BMI unavailable for three bleeding patients and 12 nonbleeding patients (five nontransfused patients and seven transfused patients). ^c^Fisher's exact test for small samples of categorical variables. APACHE, Acute Physiology and Chronic Health Evaluation; BMI, body mass index; CAD, coronary artery disease; CHF, coronary heart failure; COPD, chronic obstructive pulmonary disease; CVA, cerebrovascular accident; DM, diabetes mellitus; ESRD, end-stage renal disease; HTN, hypertension.

**Table 2:** ICU course and outcome parameters after day 21

Parameter	All patients (*n *= 155)	Never transfused (*n *= 59)	Ever transfused^a ^(*n *= 71)	*P *value
Illness acuity				
Mechanical ventilation	154 (99%)	58 (98%)	71 (100%)	0.45^b^
Inotropes/vasopressors	59 (38%)	9 (15%)	31 (44%)	0.0005
ARF requiring dialysis	24 (15%)	3 (5%)	13 (18%)	0.02
SOFA score^c^	4.8 ± 3.2	3.6 ± 2.1	5.1 ± 3.6	0.006
Anemia				
Day 22–112 Hb (g/dl)	9.1 ± 0.9	9.6 ± 1.0	8.8 ± 0.6	0.001
Phlebotomy volume (ml/pt/day)	13.3 ± 7.3	8.7 ± 4.4	14.6 ± 6.5	<0.0001
Median PRBC units transfused	1 (0–4)	-	2 (1–4)	
Outcomes				
Median ICU LOS (days)	49 (36–70)	39 (31–58)	51 (41–72)	0.001^d^
Median hospital LOS^e ^(days)	84 (55–132)	76 (55–123)	93 (56–162)	0.18^d^
ICU mortality (%)				
Days 22–112	35 (23%)	5 (8%)	16 (23%)	0.03
Total	47 (30%)	8 (14%)	23 (32%)	0.01

Values are expressed as *n *(%), mean ± standard deviation, or median (interquartile range). ^a^Excludes 25 patients with active bleeding. ^b^Fisher's exact test for small samples of categorical variables. ^c^Average score from day 21 onward; these were unavailable for 24 nonbleeding patients (12 in each group). ^d^Wilcoxon's test for non-normally distributed variables. ^e^Hospital LOS unavailable for 2 nonbleeding patients (one in each group). ARF, acute renal failure; Hb, hemoglobin; ICU, intensive care unit; LOS, length of stay; PRBC, packed red blood cells; pt, patient; SOFA, Sequential Organ Failure Assessment.
